# Shiga Toxin-Producing *Escherichia coli* Strains from Romania: A Whole Genome-Based Description

**DOI:** 10.3390/microorganisms12071469

**Published:** 2024-07-19

**Authors:** Codruța-Romanița Usein, Mihaela Oprea, Sorin Dinu, Laura-Ioana Popa, Daniela Cristea, Cornelia-Mădălina Militaru, Andreea Ghiță, Mariana Costin, Ionela-Loredana Popa, Anca Croitoru, Cristina Bologa, Lavinia-Cipriana Rusu

**Affiliations:** 1 Cantacuzino National Military Medical Institute of Research and Development, 050096 Bucharest, Romania; oprea.mihaela@cantacuzino.ro (M.O.); dinu.sorin@cantacuzino.ro (S.D.); popa.laura@cantacuzino.ro (L.-I.P.); cristea.daniela@cantacuzino.ro (D.C.); militaru.madalina@cantacuzino.ro (C.-M.M.); ghita.andreea@cantacuzino.ro (A.G.); 2 Emergency Clinical Hospital for Children “M.S. Curie”, 041451 Bucharest, Romania; marymed2006@yahoo.com (M.C.); medlore@yahoo.com (I.-L.P.); croitoru.n.anca@gmail.com (A.C.); crissoim@yahoo.com (C.B.); 3 Discipline Pediatrics—Emergency Clinical Hospital for Children M.S. Curie, University of Medicine and Pharmacy “Carol Davila” Bucharest, 050474 Bucharest, Romania; 4 National Centre for Communicable Diseases Prevention and Control, National Public Health Institute, 050463 Bucharest, Romania; lavinia.rusu@insp.gov.ro

**Keywords:** STEC, HUS, O26, WGS

## Abstract

The zoonotic Shiga toxin-producing *Escherichia coli* (STEC) group is unanimously regarded as exceptionally hazardous for humans. This study aimed to provide a genomic perspective on the STEC recovered sporadically from humans and have a foundation of internationally comparable data. Fifty clinical STEC isolates, representing the culture-confirmed infections reported by the STEC Reference Laboratory between 2016 and 2023, were subjected to whole-genome sequencing (WGS) analysis and sequences were interpreted using both commercial and public free bioinformatics tools. The WGS analysis revealed a genetically diverse population of STEC dominated by non-O157 serogroups commonly reported in human STEC infections in the European Union. The O26:H11 strains of ST21 lineage played a major role in the clinical disease resulting in hospitalisation and cases of paediatric HUS in Romania surpassing the O157:H7 strains. The latter were all clade 7 and mostly ST1804. Notably, among the Romanian isolates was a *stx2a*-harbouring cryptic clade I strain associated with a HUS case, *stx2f*- and *stx2e*-positive strains, and hybrid strains displaying a mixture of intestinal and extraintestinal virulence genes were found. As a clearer picture emerges of the STEC strains responsible for infections in Romania, further surveillance efforts are needed to uncover their prevalence, sources, and reservoirs.

## 1. Introduction

Of the infections caused by the pathogenic *Escherichia coli* strains, the ones associated with the zoonotic Shiga toxin-producing *E. coli* (STEC) group are unanimously regarded as exceptionally hazardous because of their unpredictable evolution to hemolytic uremic syndrome (HUS), a life-threatening condition with no firm solutions for prevention [1]. Moreover, their public health impact when presenting as outbreaks can be dramatic [2]. STEC members display pathogenic traits encoded by a pool of virulence and fitness genes usually located on mobile genetic elements and to assess which are the definitive ones for given strain to cause a severe disease outcome is still a medical challenge and a public health concern [3]. 

Linked to a multitude of *E. coli* serotypes and a diversity of reservoirs and vehicles, STEC infections are compulsorily monitored in most of the European Union (EU)/European Economic Area (EEA) countries which share surveillance data through the European Centre for Disease Prevention and Control (ECDC) for a supranational control and prevention of cross-border outbreaks. However, given the different ways the health systems are organised in each country and the practical issues related to the differences in the laboratory practice and recognition of pathogens, statistics may not have the same quality. Therefore, the ECDC developed a strategy for the public health system’s performance tailored on the needs and gaps of each country [4]. 

In 2016, an ECDC team conducted a visit to Romania to assist in the investigation of an outbreak identified through the geographic and time clustering of patients who had developed HUS and from whom STEC O26:H11 was isolated [5]. Until that year, the annual Romanian notification rate for STEC illness had been constantly below 0.1 cases per 100,000 population and did not exceed 6 confirmed cases notified yearly [6]. Post-outbreak, the notification rates increased (6–36 laboratory-confirmed cases/year) most likely due to an increase in the number of stool samples tested, triggered by the attention of public health authorities in combination with heightened medical awareness of HUS caused by non-O157 STEC [7]. Despite this evidence of change for the better, recognising STEC infections in the region remained a problem and the pathogens’ characteristics lacked a thorough investigation.

Herein, we aimed to provide a genomic perspective on the STEC recovered sporadically from humans after the HUS outbreak stopped to learn about the epidemiology of the infection in Romania and have a foundation of internationally comparable data. 

## 2. Materials and Methods

### 2.1. Bacterial Strains

The study was carried out in the laboratory mandated to provide national reference support for STEC identification and typing. The studied collection comprised 50 strains classified as STEC, which had been isolated between April 2016 and November 2023 from 25 patients with diarrhoea (22 children and 3 adults), 20 children with HUS, and five contacts of HUS cases with no gastrointestinal symptoms (2 children and 3 adults) (Table S1). All the strains represented sporadic cases of infections except for two pairs of strains that were recovered from matched HUS patients and contacts. 

Initially, identification and typing of STEC involved standard biochemical tests for species identification, commercial PCR-based assays for the detection of *stx1*, *stx2*, and *eae* genes, and slide agglutination tests with a commercial polyvalent (OK O pool 1–3 antisera, SSI Diagnostica, Hillerød, Denmark) and the corresponding monovalent antisera for STEC/Enteropathogenic *E. coli* (EPEC), as recommended by the manufacturer (SSI Diagnostica, Denmark). Also, *stx* subtyping was performed as described by Scheutz et al. [8]. Table S1 shows the overview of the available metadata coupled with the phenotypically derived serogroup and PCR-based genotypes for the strain collection used in this study. The strain IDs were kept as in the records of the STEC Reference Laboratory.

For comparison, for the cluster analysis and visualisation of core genomic relationships, the strains sequenced in this study were supplemented with a sequence dataset represented by the genomes of six O26:H11 [9] and three O157:H7 contextual strains [10] isolated at the beginning of 2016. Current and previous genome sequences are available under the European Nucleotide Archive (ENA) projects PRJEB37738, PRJEB37739, and PRJEB25277. The ENA-specific accession numbers for each of the genomes are listed in Table S2.

### 2.2. Whole Genome Sequencing

To perform the WGS, genomic DNA was extracted from the isolates with a High Pure PCR Template Preparation Kit (Roche Diagnostics, Indianapolis, IN, USA) or Pure Link Genomic DNA Mini kit (Invitrogen, Life Technologies Corp, Carlsbad, CA, USA) and quantified using a Qubit fluorimeter 3.0 (Life Technologies) with a Qubit dsDNA HS Assay Kit (Life Technologies). The sequence data were generated on a Life Technology Ion Torrent PGM or an Illumina NovaSeq 6000 platforms following the manufacturer’s protocols. 

For the set of 33 strains sequenced on the Ion Torrent PGM, the libraries were prepared using 400-base read length chemistry and a PGM Hi-Q View Sequencing Kit (Thermo Fisher Scientific, Waltham, MA, USA) and loaded on an Ion 318 chip v2 BC. For the 17 strains sequenced on the NovaSeq 6000 platform, the typical Illumina sequencing workflow was used with libraries prepared using an Illumina Nextera XT Library Preparation kit (San Diego, CA, USA) and the NovaSeq 6000 SP Reagent Kit v1.5 (200 cycles) for sequencing.

### 2.3. In Silico Analyses Based on Whole Genome Sequencing Data

The raw reads (FASTQ) generated by the Ion Torrent PGM were assembled with SPAdes version 3.1.0, included in Torrent Suite 5.12.1., whereas SKESA 2.4.0 integrated into Ridom SeqSphere+ version 9.0.10 (Ridom GmbH, Münster, Germany) was used for the genome assemblies in case of NovaSeq 6000 raw reads.

To assess the genetic relatedness of the strains, the FASTA assembly files were uploaded into Ridom SeqSphere+ for multilocus sequence typing (MLST) (7-allele Warwick scheme) [11] and core-genome MLST (cgMLST) (2513 alleles) [12]. A minimum spanning tree (MST) based on the cgMLST allelic profiles was constructed using SeqSphere+ with the option “pairwise ignoring missing values” turned on and the single-linkage threshold of ≤1 allele maximum distance for cluster alert. Also, genome assemblies were uploaded into the public Center for Genomic Epidemiology (CGE) server https://cge.cbs.dtu.dk/services/ (accessed on 20 June 2023) to extract further information on the serotype, virulence genes, antimicrobial resistance genes, and plasmid replicons with SerotypeFinder 2.0 [13], VirulenceFinder 2.0 [14,15], ResFinder 4.1 [16,17], and PlasmidFinder 2.1 tools [17,18], respectively, for which the settings were left at default values. The ClermonTyping method and its associated web-based interface http://clermontyping.iame-research.center (accessed on 7 May 2023) were used for phylotyping [19]. 

Clade typing of the O157:H7 strains relied on the identification of clade-specific SNPs, originally described by Manning et al. [20], targeting known informative positions [21].

Also, the Simpson’s diversity index (DI) was calculated using the WGS-based serotype data and the following equation:

$$DI=1-[\frac{1}{N\left(N-1\right)}]\sum_{j=1}^{s}n_{j}(n_{j}-1)$$

where *s* is the number of serotypes, *n* is the number of strains belonging to a single serotype, and N is the total number of strains [22].

## 3. Results

### 3.1. Correlation between Serotypes, Clinical Status, Sequence Types, Phylogroups, and Virulence Genes

Ion Torrent and Illumina raw reads obtained ranges between 1,003,793–2,815,936 and 1,998,316–9,633,052 reads, respectively. The predicted genome sizes of the 50 strains ranged from 4.6 Mb to 5.7 Mb with between 114 and 780 contigs (>200 bp) and at least 37× depth of coverage (Ion Torrent PGM/38.5×–143.2× coverage, NovaSeq6000/37×–174× coverage). The highest N50 value was 186,789 bp while the lowest N50 value was 25,638 bp (Table S2).

The SerotypeFinder generated full O and H antigen information for 48 genomes, confirming all the initially O-typeable strains reported based on the conventional slide agglutination. Two genomes with good-quality assemblies were deemed O-nontypeable (Ont) as there was no antigen O prediction. In total, 15 different O-groups (O9a, O10, O26, O43, O55, O76, O78, O91, O100, O104, O113, O125ac, O128ac, O146, and O157) and 12 H-types (H2, H4, H6, H7, H10, H11, H12, H14, H19, H20, H21, and H45) were predicted. Their combinations generated 17 distinctive serotypes (including the Ont:H types) and a DI value of 0.738. There were five multiple-strain serotypes (i.e., O26:H11/25 strains, O157:H7/6 strains, O113:H4/3 strains, O91:H14/2 strains, and O128ac: H2/2 strains), which accounted for 76% of the collection, and 12 single-strain serotypes (i.e., O146:H2, O125ac: H6, O104:H21, O100:H20, O78:H4, O76:H19, O55:H12, O43:H2, O10:H45, O9a:H10, Ont:H4, and Ont:H10). 

The 21 HUS cases were correlated to five serotypes, namely O26:H11, O104:H21, O10:H45, Ont:H4, and Ont:H10, but the O26:H11 strains were responsible for most of them (81%). In the diarrhoea cases, ten serotypes were involved, of which O26:H11 and O157:H7 accounted for 50% of them. As for the strains carried asymptomatically, they belonged to the O26:H11, O91:H14, O43:H2, and O76:H19 serotypes.

By using the ClermonTyper tool, 49 of 50 presumptive STEC strains were verified as *E. coli sensu stricto*, whereas a strain assigned to the *E. coli* serotype O10:H45 was placed in the *Escherichia* cryptic clade I (CI). Among the *E. coli* strains, an inconsistent phylogroup assignment was noted for an O9a:H10 strain which was A phylogenetic group by in silico Clermont typing and in B1 by Mash analysis. A further NCBI BLAST (https://blast.ncbi.nlm.nih.gov/Blast.cgi, accessed on 7 May 2023) search was performed to explain this discordance. The TspE4.C2 marker specific for the phylogenetic group B1 strains was not detected in the analysed genome. Also, EnteroBase assigned this strain to phylogenetic group A and identified two other strains belonging to this phylotype at no more than 200 alleles (HC200) apart, and the other at no more than a 400-allele distance (HC400) [23]. Therefore, we decided to assign the strain to phylogenetic group A. However, it did not significantly change the phylogroup representation, which, according to the in silico PCR, was dominated by phylogroup B1 followed at a distance by phylogroup A. Specifically, there were 43 phylogroup B1 strains representing eight serotypes (O26:H11, O43:H21, O55:H12, O76:H19, O91:H14, O104:H21, O128ac:H2, and O146:H21) and 7 phylogroup A assigned to five serotypes (O9a:H10, O100:H20, O113:H4, Ont:H4, and Ont:H10). The collection also included six phylogroup E strains, all belonging to the O157:H7 serotype, one phylogroup B2 O125ac:H6 strain, and a representative of phylogroup C assigned to the O78:H4 serotype.

The MLST analysis distinguished 17 distinctive sequence types (STs), which, combined with the strains’ serotypes, resulted in 19 ST/serotype combinations (Table 1). The serotype O26:H11 strains represented ST21 and ST15140, a new ST with *fumC* 961 allele. Also, the O157:H7 strains belonged to ST11 and the single-locus variant (SLV) ST1804. The ST10 lineage was represented by the O113:H4 strains and an Ont:H4 strain. The non-*E. coli* isolate was typed as ST10716.

The use of the VirulenceFinder tool enabled the detection of 69 markers related to *E. coli* virulence apart from the *stx* and *eae* genes. Twenty-two of them were found in at least 50% of the strains (i.e., *cif*, *efa1*, *ehxA*, *espA*, *espB*, *espF*, *espJ*, *espP*, *fyuA*, *gad*, *hlyE*, *iha*, *irp2*, *iss*, *katP*, *lpfA*, *nleB*, *nleC*, *ompT*, *terC*, *tir*, and *traT*), 20 in 10–48% of the strains (i.e., *cba*, *colE9*, *cia*, *chuA*, *cma*, *espI*, *etpD*, *ireA*, *iucC*, *iutA*, *kpsE*, *mchB*, *mchC*, *mchF*, *nleA*, *senB*, *sitA*, *subA*, *tccP*, and *toxB*) and 27 in no more than 8% of the strains (i.e., *aaiC*, *aalF*, *afaA*, *afaB*, *afaC*, *afaD*, *afaE8*, *air*, *astA*, *cdtB*, *cea*, *cib*, *cnf2*, *ctaA*, *cvaC*, *eilA*, *epeA*, *espC*, *estap*, *estb*, *hlyA*, *ibeA*, *neuC*, *papC*, *pic*, *sepA*, and *yfcV*). 

Concerning the Shiga toxin-encoding genes, there was exact concordance between the *stx* types and subtypes found using routine PCR-based typing and WGS. Therefore, nine strains harboured *stx1* gene alone, 29 possessed *stx2* alone, and 12 strains harboured a combination of *stx1* and *stx2*. The *stx1* genes found were *stx1a* (14 strains) and *stx1c* (7 strains). The following *stx2* subtypes were identified: *stx2a* (22 strains), *stx2b* (9 strains), *stx2c* (6 strains), *stx2d* (one strain), *stx2e* (2 strains), and *stx2f* (one strain). The subtype combinations found were *stx1c* + *stx2b* (5 strains), *stx1a* + *stx2a* (2 strains), *stx1a* + *stx2b* (2 strains), *stx1a* + *stx2c* (2 strains), and *stx1a* + *stx2d* (1 strain) (Table 1).

The WGS analysis confirmed the presence of the *eae* gene in all the 32 *eae*-PCR positive strains, identifying three gene alleles, *eae-α* (one strain), *eae-β* (25 strains), and *eae-γ* (6 strains).

To quantify the virulence of a strain or a group of strains, virulence scores (VSs) were computed ad hoc, corresponding to the total number of WGS-inferred virulence genes per strain and the aggregate number of virulence genes divided by the number of group’s strains, respectively. The VS value per strain ranged from 6 to 30 (average VS = 22) and correlated with 42 gene combinations or profiles (VPs) with a DI value of 0.991. 

The *eae*-positive strain group, representing 3 serotypes, gathered 40 of the 78 virulence genes identified in this study which were displayed in 24 different VPs (DI = 0.979) with an average VS of 25. Within this group, the O26:H11 strains harboured the highest number of *E. coli* virulence-associated gene markers (average VS = 26) combined in 18 VPs with a common core of 15 genes (i.e., *cif*, *eae*, *efa1*, *ehxA*, *espA*, *espB*, *espF*, *espP*, *irp2*, *iss*, *lpfA*, *ompT*, *terC*, *tir*, and *traT*) and the following *stx* subtypes/combinations: *stx1a* (6 strains), *stx2a* (17 strains) and *stx1a* + *stx2a* (2 strains). Many strains also harboured *katP*, *hlyE*, *espJ*, *nleB*, *nleC*, and *fyuA* genes. The O157:H7 serotype had a lower VS than the O26:H11 (average VS = 22), being represented by strains devoid of genes unanimously present in the O26:H11 strains such as *cif*, *efa1*, *espF*, *irp2*, and *lpfA* while harbouring *chuA*, *etpD*, *hlyA*, and *stx2c* instead. The *stx* subtypes/combinations linked to this serotype were *stx2c* alone (4 strains) and *stx1a* + *stx2c* (2 strains). The lowest VS was computed for the *stx2f*-harbouring O125ac:H6 strain with 12 virulence markers, *cif*, *chuA*, *eae*, *espA*, *espC*, *gad*, *ibeA*, *ompT*, *terC*, *tir*, and *yfcV*.

The group of strains which lacked the *eae* gene, assigned to 14 serotypes, had VS values between 22 and 6 (average VS = 16) resulting from the various combinations of 57 genes out of the 78 detected (DI = 1). Overall, between 38% and 50% of the strains possessed *ireA*, *kpsE*, *mchB*, *mchC*, *mchF*, *senB*, *stx1c*, *stx2b*, and *subA* genes, but the diversity of their VPs was mostly due to genes found in at most two strains each (4%). Among these rare genes were *afa* (i.e., *afaA*, *afaB*, *afaC*, *afaD*, and *afaE8*) and *cnf2* found in the O55:H12 strain, the *stx2e* gene detected in the strains assigned to the serotypes O9a:H10 and O100:H20; the *estap*, *estb*, and *sepA* genes were also harboured by the O100:H20 strain, the *air*/*eaeX* and *eilA* genes present in the *stx2a*-positive O10:H45 strain was presumed to belong to the CI. 

When considering the virulence gene content of the multiple-strain serotypes, the O128ac:H2 serotype was the richest with 25 virulence markers, followed by O91:H14 and O113:H4 with 22 and 18 genes, respectively. As for the collection’s serotypes, represented by single strains, O146:H2 and O76:H19 possessed the highest VS values (i.e., VS = 21), as opposed to the O9a:H10 strain which had the lowest number of virulence markers (VS = 6).

The information regarding the diversity of serotypes, sequence types, phylogenetic groups, antimicrobial resistance genotypes, and virulence gene profiles characteristic of the autochthonous *E. coli* strains included in this study is presented in Table S3. 

### 3.2. Core Genome MLST-Derived Relatedness of O26:H11 Strains 

**Figure 1:**
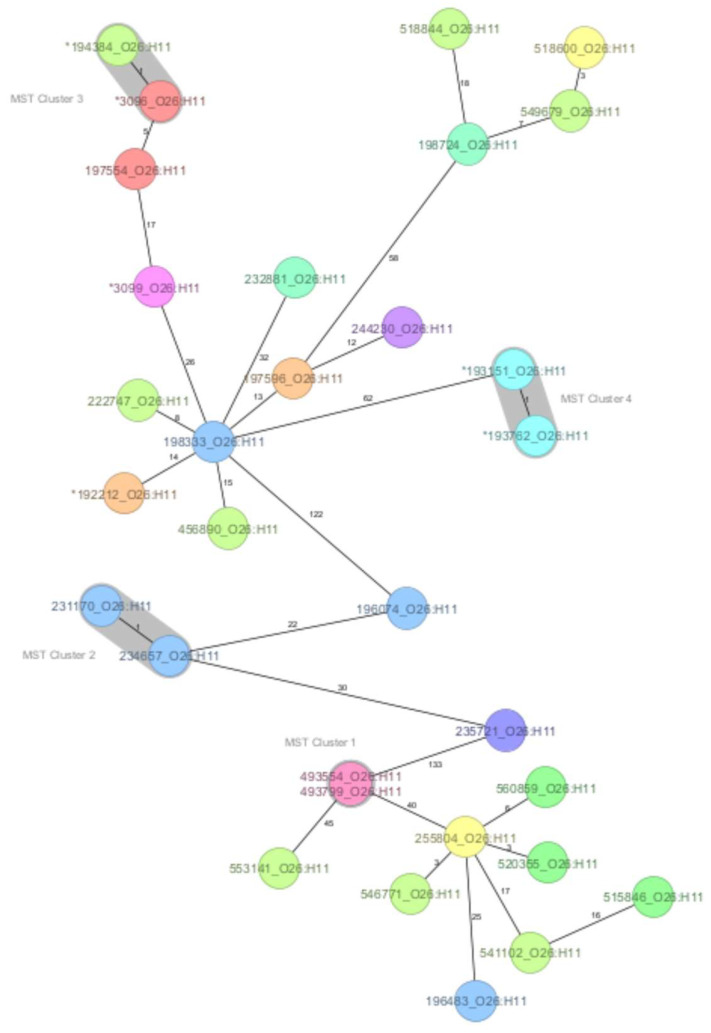
The minimum spanning tree (MST) of the Shiga toxin-producing *E. coli* (STEC) serotype O26:H11 Romanian strains analysed in this study (historical strains included), based on core-genome multilocus sequence type allelic profiles. The analysis and tree were obtained with the public Ridom-SeqSphere+-integrated *E. coli* cgMLST scheme of 2513 target loci. On the tree, allele distances between samples are indicated. The different colours are related to the Romanian counties of strains’ origin. Clusters of samples with one allele distance are shaded in grey. The asterisk (*) placed before the strain ID denotes a historical strain.

A core genome MLST was used to assess the genetic relatedness within the O26:H11 and O157:H7 serotypes, as the strains were sufficient in number to support a more detailed analysis, and historical strains were available for comparison. The MST generated for the O26:H11 serotype showed that the maximum number of allelic differences (AD) was 133. At the one-AD threshold, four clusters were defined with two strains each. Cluster 1 was formed by strains with identical allelic profiles and known epidemiological links that originated from siblings, one with HUS and the other asymptomatic. Cluster 2 indicated two strains from this study that shared marked genomic similarity but had no apparent epidemiological link, representing HUS cases reported two months apart in neighbouring counties. Clusters 3 and 4 confirmed the close genomic relationship of strains with the 2016 HUS outbreak provenance (Figure 1). 

**Figure 2:**
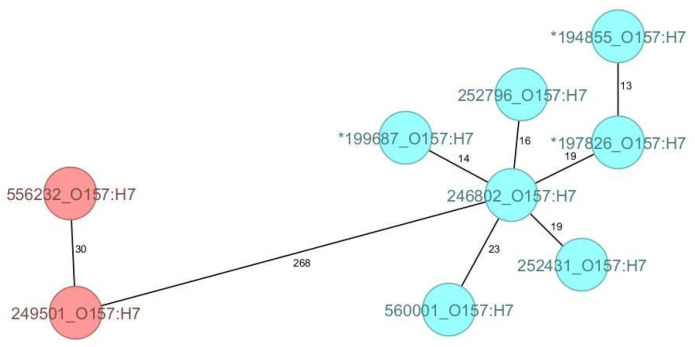
The minimum spanning tree (MST) of the Shiga toxin-producing *E. coli* (STEC) serotype O157:H7 Romanian strains analysed in this study (historical strains included), based on core-genome multilocus sequence type allelic profiles. The analysis and tree were obtained with the public Ridom-SeqSphere+-integrated *E. coli* cgMLST scheme of 2513 target loci. On the tree, allele distances between samples are indicated. The different colours are related to the sequence types, with red indicating ST11 and blue ST1804. The asterisk (*) placed before the strain ID denotes a historical strain.

As for the O157:H7 strains compared in this study, all belonged to clade 7 and, although genetically diverse, they clustered according to their ST, forming two discrete groups with 268 AD (Figure 2). 

### 3.3. Antimicrobial Resistance and Plasmid Content

**Figure 3:**
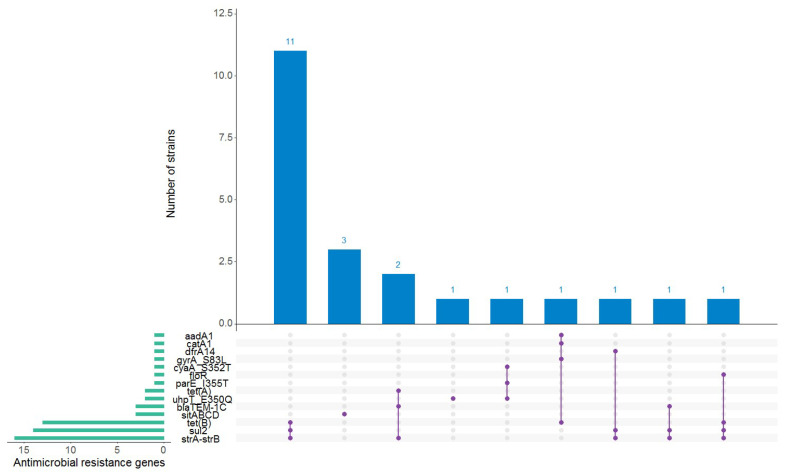
Antimicrobial resistance (AMR) genes found in the autochthonous Shiga toxin-producing *Escherichia coli* strains. The horizontal green bars represent the frequency of each antimicrobial resistance gene. An AMR genotype is represented by the purple linked dots. The blue vertical bars represent the number of strains positive for a particular AMR genotype. This figure was generated using RStudio version 1.2.5001 [24] with the UpSetR package [25].

When the genome sequences were interrogated for genes and point mutations known to confer resistance to the antibiotics present in the ResFinder database, 22 of the 50 strains (44%) carried between one and four antimicrobial resistance (AMR) genes whose presence was predicted to confer resistance to aminoglycosides (*aadA1*, *strA-strB*), sulfonamides (*sul2*), tetracyclines (*tetA*, *tetB*), beta-lactams (*blaTEM-1C*), folat synthesis inhibitors (*dfrA14*), phenicols (*floR*, *catA1*), quinolones (*gyrA_*S83L, *parE_*I355T), fosfomycin (*uhpT*_E350Q, *cyaA*_S352T), and disinfectants (*sitABCD*) (Figure 3). The highest proportion of strains predicted to be resistant was observed within the O26:H11 serotype, which were assigned the following AMR genotypes: *strA/strB* + *tetB* + *sul2* (11 strains), *blaTEM-1C* + *straA/strB* + *tetA* (2 strains), *blaTEM-1C* + *straA/strB* + *sul2* (one strain), and *strA/strB* + *dfrA14* + *sul2* (one strain). The remaining strains that carried AMR genes belonged to the following O10:H45 (one strain), O91:H14 (2 strains), O125ac:H6 (one strain), O157:H7 (one strain), O128ac:H2 (one strain), and O157:H7 (one strain), respectively (Table S3).

PlasmidFinder indicated the presence of plasmid replicon sequences from 27 different incompatibility groups among the 50 strains with between one and six replicon types per strain. The most frequently harboured replicons were IncFIB (AP001918) (44 strains) followed by IncB/O/K/Z sequences (27 strains), IncFII(pCoo) (10 strains), IncFII (9 strains), and Col156 (9 strains). In particular, all the O26:H11 strains carried, concurrently, IncFIB (AP001918) and IncB/O/K/Z sequences, whereas the O157:H7 strains harboured IncFIB (AP001918) with IncFIC(FII) instead. Also, IncFII(pCoo) and Col156 were found mostly in the strains from the *eae*-negative group.

## 4. Discussion

This study re-examined the STEC isolates identified by the STEC Reference Laboratory over eight years hoping that the genomic insights into these pathogens will be of public health use for Romania as part of Europe. The strains derived from routine diagnostic activities performed between 2016 and 2023, after the HUS outbreak was over, and were representing mostly paediatric cases, a finding raising the question of whether this overrepresentation of children was related to increased exposure, susceptibility, or diagnostic efforts [26,27]. 

As the collection studied comprised almost eight times as many non-O157 as O157 strains, and 43% of them had no phenotypically derived serogroup, this study provided data to broaden the knowledge of the diversity and prevalence of STEC serotypes in human disease in Romania. Hence, the serotyping data, predicted by the SerotypeFinder tool with 96% sensitivity for O-antigens and 100% for H-antigens, revealed a collection with a moderate serotype diversity, comprising representatives of 17 serotypes of at least 15 serogroups. We say at least because two strains had incomplete antigen profiles with no O-antigen predicted despite the good quality of read data. Studies evaluating the open-source tools for in silico serotyping, including the one used in this study, offered possible explanations for the failures observed in their performance while also concluding that further benchmarking and comparison studies are needed [28,29]. Meanwhile, the information about the STEC serotypes in circulation in Romania was already superior to the historical data biased by the traditional agglutination approach, evidencing that the serogroups commonly reported in human STEC infections in the European Union between 2016 and 2019, such as O157, O26, O55, O76, O78, O91, O113, O128, and O146, were also clinically relevant in Romania [30,31].

In the diversity of serotypes captured in the studied timespan, five serotypes appeared more than once, O26:H11, O157:H7, O113:H4, O128ac:H2, and O91:H14, reflecting most likely epidemiological features specific to the region which are difficult to clarify retrospectively because there are many knowledge gaps in the epidemiology of STEC in Romania to be filled before one can fully understand the public health impact of the STEC threat. However, it is indisputable that since the stability in its circulation since 2016, when it emerged as a public health threat, the O26:H11 serotype has played a major role in the clinical disease, resulting in hospitalisation and cases of paediatric HUS in Romania. Therefore, for the moment, the serotype results were suitable to illustrate that Romania was not spared from the emerging risk of infections caused by STEC serogroups other than O157 and the increasing number of severe STEC O26 cases observed in recent years in many countries was evident in this country as well [26,32]. 

All the Romanian STEC O26:H11 strains described in this study were assigned to ST21 except for one, typed as an SLV of ST21, and harboured only *stx1a* and *stx2a* subtypes, with *stx2a*-positive strains almost three times more prevalent than those with a *stx1*-positive genotype. As expected, in line with the specific characteristics delineating the ST21 lineage, they uniformly displayed the typical plasmid gene profile described for ST21 lineage, consisting of the presence of the genes encoding enterohemolysin (*ehxA*), catalase–peroxidase (*katP*), and serine protease (*espP*) and the absence of the gene coding for an effector of the type II protein secretion system (*etpD*) [33]. Put together with the central possession of *cif*, *eae-β*, *efa1*, *ehxA*, *espA*, *espB*, *espF*, *espJ*, *espP*, *fyuA*, *hlyE*, *irp2*, *iss*, *katP*, *lpfA*, *nleB*, *nleC*, *ompT*, *terC*, *tir*, and *traT* genes, these traits were considered broadly characteristic of the O26:H11 strains circulating in Romania between 2016 and 2023 and supported the conclusion that the ST21 clone is endemic in Romania while ST29 has not emerged yet, despite the broad spread of such STEC throughout Europe and beyond [34,35].

Cattle are regarded as a main reservoir of STEC O26:H11 strains [36,37]. A large-scale genomic analysis showed that the bovine and human isolates are phylogenetically indistinguishable, at least in the ST21 lineage [35]. Starting from this, we speculate on the presence of a large livestock source of STEC O26:H11 ST21 in Romania acknowledging that further studies are needed to document consistently the sources and reservoirs of STEC in our country as there is no national surveillance programme to investigate STEC in cattle or other animals at a national level. Although scarce, the data available from the veterinary side support our hypothesis, adding the worrying aspect of antibiotic resistance in animal and food products. A retrospective study of *E. coli* strains isolated from diseased young calves between 1980 and 2016 reported STEC serogroups also described among humans, such as O26, O78, and O146. A significant increase in the number of strains with multidrug resistance phenotypes and particularly with co-resistance to tetracycline, sulphonamides and streptomycin were also reported for the period 2000–2016 [38]. STEC was also reported as present in the locally produced raw milk and unpasteurized traditional cheeses marketed in 2014 in Romania, the products posing a high human risk of contamination with strains harbouring genes incriminated for resistance to at least one antibiotic and mostly to tetracycline [39]. Additionally, in a recent study carried out in two large abattoirs from Romania and France, a 17% STEC prevalence was reported for each, and 68% multidrug-resistant strains were isolated in Romania compared to 38% in France [40]. In this context, the high prevalence of resistance determinants against sulphonamides and tetracyclines, particularly among the human strains of the prominent O26:H11 serotype, with 44% of strains harbouring the combination of resistance determinants against aminoglycosides (*strA-strB*), sulphonamides (*sul2*), and tetracyclines (*tetB*) showed that the subject of cattle as reservoirs of antibiotic-resistance genes for humans deserves the same attention locally as internationally [41].

In this study, all the STEC non-O157 serotypes were represented by phylogenetically diverse strains derived from phylogenetic groups A, B1, B2, C, and cryptic CI. The detection of the latter was notable since, to our knowledge, it is the first evidence of the association of cryptic clade members with human disease in Romania, and more precisely with HUS. The strain predicted to be of the rare serotype O10:H45 carried the *stx2a* with other genes encoding intestinal and extraintestinal virulence factors described among the human and animal *E. coli* isolates. Its virulence gene profile included the *eilA* (encoding a regulator) and *air*/*eaeX* (encoding enteroaggregative immunoglobulin-repeat protein) genes, which were originally described in the enteroaggregative *E. coli* (EAEC) 042 strain, as located on the *eip* genomic island [42]. There were also the plasmid-encoded genes *iss* (encoding increased serum survival), *traT* (encoding an outer membrane lipoprotein), and *hlyA* (encoding alpha-hemolysin), as well as the *lpfA* gene coding for an adhesin (long polar fimbriae) associated with STEC of various serotypes [43,44,45]. Although only a sporadic case was linked to a clinically relevant CI strain in this study, its detection raised the question about the autochthonous reservoirs and prevalence of these divergent *E. coli* lately regarded as an emerging source of human intestinal pathogens carrying STEC and Enterotoxigenic *E. coli* (ETEC) virulence genes [46]. 

Before this study, due to limited resources and time constraints, the Reference Laboratory performed the PCR-based *stx* subtyping of STEC strains in batches now and then. Therefore, the scrutiny of the *stx2* and *eae* combination performed for the diagnosis of STEC infections was denoted as suitable to predict potentially severe outcomes, the latter gene used as the main indicator of the chromosomal Locus of Enterocyte Effacement (LEE) pathogenicity island harboured commonly by strains from hypervirulent lineages [47,48,49]. The WGS-based characterisation verified the information utilised in assessing the potential health risk of the STEC strains for eight years while augmenting it to a level that would not have been possible before, putting the autochthonous STEC infections in a new epidemiological perspective. 

The non-O157:H7 group of strains illustrated best what is known about certain toxin subtypes being more closely associated with severe clinical outcomes than others and the occurrence of more pathogenic lineages through the acquisition and expression of new genes in a specific genetic background [50,51]. If disregarding the prominent O26:H11 subset, this group comprised almost exclusively LEE-negatives, which harboured diverse variants of Shiga toxin-encoding genes. Although these strains were more often associated with non-complicated diarrhoea, a handful of them were responsible for the host progression to HUS, highlighting, if still necessary, the current public health opinion that all STEC strains should be considered pathogenic in humans [3]. Notably, one of the strains associated with HUS belonged to the O104:H21 serotype and ST672 lineage and carried *stx1a*, *stx2d*, and *ehxA*, similar to previously described STEC O104:H21 isolates responsible for an outbreak of haemorrhagic colitis in the United States of America [52] or associated with HUS in Germany [53]. The others were two *stx2a*-harbouring strains belonging to Ont:H4 and STEC Ont:H10 serotypes which encoded characteristics of EAEC (*aaiC*) [54] and Diffusely Adherent *E. coli* (*afaD*) pathotypes [55]. 

More than half (59%) of the strains associated with milder illness carried *stx2b* alone or associated with *stx1a* or *stx1c* and represented five serotypes, O76:H19, O91:H14, O113:H4, O128ac:H2, and O146:H2. Starting from this, we can presume that at least the multiple-strain clones, O91:H14, O113:H4, and O128ac:H2, may well have been spread more but the less severe accompanying symptomatology lowered the addressability to medical services and diminished the number of stool cultures for such strains. The *stx2f* and *stx2e* subtypes commonly detected in the STEC strains of animal origin and rarely in human isolates were also associated with STEC-induced diarrhoea, and this study was the first to report the genetic makeup of such strains in Romania. The *stx2f*-positive strain belonged to the O125ac:H6 serotype, clonal complex 122/ST583, and phylogroup B2 and carried core LEE genes (*eae-alpha*, *espA*, *tir*), non-LEE genes (*cif*, *espC*), and virulence determinants linked to the extraintestinal pathogenicity (*chuA*, *ibeA*, *ompT*, *yfcV*). Similar characteristics have been previously reported for a human isolate in an Italian study comparing Stx2f-producing STEC strains isolated from humans and pigeons, the latter considered a potential reservoir for these pathogens [56]. Furthermore, there was an increasing number of infections caused by STEC-harbouring *stx2f* of various serotypes/lineages, including he O125ac:H6/ST583,notified in England between 2015 and 2022, but there were no specific animal or environmental reservoirs exposed [57]. 

The *stx2e*-positive *E. coli* strains originated from subjects with no epidemiological links. Similar to other strains lysogenized with bacteriophages carrying the *stx2e* gene, these strains were LEE-negative and lacked the *ehxA* gene (encoding enterohemolysin) [58]. One of the strains belonged to the O9a:H10 serotype which denoted an O antigen with high homology to *Klebsiella pneumoniae* O3 polysaccharides [59]. Its virulence gene profile was attributed the lowest virulence score in this study and consisted in a combination of low-prevalent genes, such as *astA* (encoding *E. coli* heat-stable enterotoxin), *cea* (encoding colicin E1), and *sitA* (encoding an iron-transport protein), and genes with a broad distribution across the autochthonous STEC, such as *terC* (encoding tellurium ion resistance protein) and *traT* (encoding outer-membrane protein complement resistance). The O100:H20 strain typed ST2514 qualified as a hybrid Shiga toxigenic and ETEC based on the possession of *estap* (encoding heat-stable enterotoxin STa1 porcine variant), *estb* (encoding heat-stable enterotoxin STb1), and *sepA* (encoding serine protease SepA autotransporter) genes. Such strains, with STEC/ETEC status belonging to the O9 and O100, serogroups have been reported with high prevalence in swine [60] and the O100:H20 serotypes specifically were associated with both healthy pigs and wild boars, the latter suspected of transmission events at the wildlife–livestock interface but was also reported as clinically relevant [61,62].

It should be mentioned that many of the strains investigated in this study displayed virulence genes registered in the VirulenceFinder database as related to extraintestinal pathogenicity which is in line with previous reports on *E. coli* hybrids displaying combinations of intestinal and extraintestinal virulence found frequently, now that genome sequence analyses enable their identification [63]. Notably, the highest number of hybrids was found among the LEE-negative strains. Such a peculiar strain was the *stx1a*-harbouring O55:H12 strain assigned to ST101 and phylogenetic group B1, which contained the genes coding for the cytotoxic necrotizing factor type 2, previously detected in *E. coli* strains associated with various animals, diseases, and genes of the *afa* operon, coding for afimbrial adhesins involved in both diarrhoea and extraintestinal infections in animals and humans [64,65]. Furthermore, the association of *cnf2* and *afaE8* suggested the animal origin of this strain [66]. 

In Europe, despite the continuously increasing proportion of non-O157 STEC serogroups observed since the start of serogroup surveillance, particularly in the O26 serogroup, STEC O157 remained the most frequently reported serogroup in the confirmed cases [26]. As mentioned, O157:H7 had a far lower detection rate than the O26:H11 serotype in Romania and less clinical relevance as well, if considering that the strains collected from 2016 to 2023 originated exclusively from uncomplicated cases of diarrhoea. The epidemiology of STEC infections is changing, influenced by the genetic variation in the population of strains, and this was remarked for the O157:H7 clone as well. The molecular approaches developed to address the virulence differences among the O157:H7 strains delineated nine clades [20,21]. The autochthonous O157:H7 human isolates were split by an in silico MLST into ST11 and ST1804, the latter apparently more spread in the country, and were typed clade 7. Their *stx* genotypes, *stx2c* alone or in combination with *stx1a*, and clinical presentation correlated with this clade, which defines the strains that were less associated with HUS [20]. 

## 5. Conclusions

This study was meant to be a forerunner of the WGS implementation into routine practise at the national level and provide a reference genomic database of DNA sequences for interrogation. As a clearer picture emerges on the STEC strains responsible for infections in Romania, further surveillance efforts are needed to uncover their prevalence, sources, and reservoirs and support decisions on public health interventions for STEC infections.

## Figures and Tables

**Table 1:** Summary of serotypes, sequence types, and Shiga toxin gene (*stx*) genotypes in dataset.

Serotype	Sequence Type	*stx* Genotype	No. of Strains
O26:H11	ST21; ST15140	*stx1a*; *stx2a*; *stx1a* + *stx2a*	25
O157:H7	ST11; ST1804	*stx2c*; *stx1a* + *stx2c*	6
O113:H4	ST10	*stx1c* + *stx2b*	3
O91:H14	ST33	*stx1a* + *stx2b*	2
O128ac:H2	ST25	*stx2b*	2
O9a:H10	ST1791	*stx2e*	1
O10:H45	ST10716	*stx2a*	1
O43:H2	ST937	*stx1c*	1
O55:H12	ST101	*stx1a*	1
O76:H19	ST675	*stx1c* + *stx2b*	1
O78:H4	ST3101	*stx1c*	1
O100:H20	ST2514	*stx2e*	1
O104:H21	ST8160	*stx1a* + *stx2d*	1
O125ac:H6	ST583	*stx2f*	1
O146:H2	ST442	*stx1c* + *stx2b*	1
Ont:H4	ST10	*stx2a*	1
Ont:H10	ST34	*stx2a*	1

Note: Ont, O antigen nontypeable.

## Data Availability

The raw data supporting the conclusions of this article will be made available by the authors on request.

## Supplementary Materials

The following supporting information can be downloaded at: https://www.mdpi.com/article/10.3390/microorganisms12071469/s1, Table S1: Isolates sequenced in this study and the associated data, Table S2: Whole genome sequencing experiment information, and Table S3. Serotypes, sequence types, phylogroups, and virulence genes of the samples included in dataset based on WGS.

## References

[1] Mühlen S., Dersch P. (2020). Treatment Strategies for Infections with Shiga Toxin-Producing *Escherichia coli*. Front. Cell Infect. Microbiol..

[2] Radosavljevic V., Finke E.J., Belojevic G. (2015). *Escherichia coli* O104:H4 outbreak in Germany—Clarification of the origin of the epidemic. Eur. J. Public Health.

[3] Koutsoumanis K., Allende A., Alvarez-Ordóñez A., Bover-Cid S., Chemaly M., Davies R., De Cesare A., Herman L., Hilbert F., EFSA BIOHAZ Panel (2020). Scientific Opinion on the pathogenicity assessment of Shiga toxin-producing *Escherichia coli* (STEC) and the public health risk posed by contamination of food with STEC. EFSA J..

[4] https://www.ecdc.europa.eu/en/publications-data/amended-ecdc-strategy-2021-2027.

[5] Severi E., Vial F., Peron E., Mardh O., Niskanen T., Takkinen J. (2016). Community-wide outbreaks of haemolytic uraemic syndrome associated with Shiga toxin-producing *Escherichia coli* O26 in Italy and Romania: A new challenge for the European Union. Eurosurveillance.

[6] European Centre for Disease Prevention and Control (2018). Shigatoxin/verocytotoxin-producing *Escherichia coli* (STEC/VTEC) infection. Annual Epidemiological Report for 2015.

[7] European Centre for Disease Prevention and Control (2022). STEC infection. Annual Epidemiological Report for 2021.

[8] Scheutz F., Teel L.D., Beutin L., Piérard D., Buvens G., Karch H., Mellmann A., Caprioli A., Tozzoli R., Morabito S. (2012). Multicenter evaluation of a sequence-based protocol for subtyping Shiga toxins and standardizing Stx nomenclature. J. Clin. Microbiol..

[9] Usein C.R., Ciontea A.S., Militaru C.M., Condei M., Dinu S., Oprea M., Cristea D., Michelacci V., Scavia G., Zota L.C. (2017). Molecular characterisation of human Shiga toxin-producing *Escherichia coli* O26 strains: Results of an outbreak investigation, Romania, February to August 2016. Eurosurveillance.

[10] Oprea M., Ciontea A.S., Militaru M., Dinu S., Cristea D., Usein C.R. (2018). Molecular Typing of *Escherichia coli* O157 Isolates from Romanian Human Cases. Jpn. J. Infect. Dis..

[11] Wirth T., Falush D., Lan R., Colles F., Mensa P., Wieler L.H., Karch H., Reeves P.R., Maiden M.C., Ochman H. (2006). Sex and virulence in *Escherichia coli*: An evolutionary perspective. Mol. Microbiol..

[12] Zhou Z., Alikhan N.F., Mohamed K., Fan Y., Achtman M., Agama Study Group (2020). The EnteroBase user’s guide, with case studies on *Salmonella* transmissions, *Yersinia pestis* phylogeny, and *Escherichia* core genomic diversity. Genome Res..

[13] Joensen K.G., Tetzschner A.M., Iguchi A., Aarestrup F.M., Scheutz F. (2015). Rapid and Easy In Silico Serotyping of *Escherichia coli* Isolates by Use of Whole-Genome Sequencing Data. J. Clin. Microbiol..

[14] Joensen K.G., Scheutz F., Lund O., Hasman H., Kaas R.S., Nielsen E.M., Aarestrup F.M. (2014). Real-time whole-genome sequencing for routine typing, surveillance, and outbreak detection of verotoxigenic *Escherichia coli*. J. Clin. Microbiol..

[15] Malberg Tetzschner A.M., Johnson J.R., Johnston B.D., Lund O., Scheutz F. (2020). In Silico Genotyping of *Escherichia coli* Isolates for Extraintestinal Virulence Genes by Use of Whole-Genome Sequencing Data. J. Clin. Microbiol..

[16] Bortolaia V., Kaas R.S., Ruppe E., Roberts M.C., Schwarz S., Cattoir V., Philippon A., Allesoe R.L., Rebelo A.R., Florensa A.F. (2020). ResFinder 4.0 for predictions of phenotypes from genotypes. J. Antimicrob. Chemother..

[17] Camacho C., Coulouris G., Avagyan V., Ma N., Papadopoulos J., Bealer K., Madden T.L. (2009). BLAST+: Architecture and applications. BMC Bioinform..

[18] Carattoli A., Zankari E., Garcia-Fernandez A., Voldby Larsen M., Lund O., Villa L., Aarestrup F.M., Hasman H. (2014). PlasmidFinder and pMLST: In silico detection and typing of plasmids. Agents Chemother..

[19] Beghain J., Bridier-Nahmias A., Le Nagard H., Denamur E., Clermont O. (2018). ClermonTyping: An easy-to-use and accurate in silico method for *Escherichia* genus strain phylotyping. Microb. Genom..

[20] Manning S.D., Motiwala A.S., Springman A.C., Qi W., Lacher D.W., Ouellette L.M., Mladonicky J.M., Somsel P., Rudrik J.T., Dietrich S.E. (2008). Variation in virulence among clades of *Escherichia coli* O157:H7 associated with disease outbreaks. Proc. Natl. Acad. Sci. USA.

[21] Riordan J.T., Viswanath S.B., Manning S.D., Whittam T.S. (2008). Genetic differentiation of *Escherichia coli* O157:H7 clades associated with human disease by real-time PCR. J. Clin. Microbiol..

[22] Hunter P.R., Gaston M.A. (1988). Numerical index of the discriminatory ability of typing systems: An application of Simpson’s index of diversity. J. Clin. Microbiol..

[23] Zhou Z., Charlesworth J., Achtman M. (2021). HierCC: A multi-level clustering scheme for population assignments based on core genome MLST. Bioinformatics.

[24] RStudio Team (2019). RStudio: Integrated Development for R.

[25] Conway J.R., Lex A., Gehlenborg N. (2017). UpSetR: An R package for the visualization of intersecting sets and their properties. Bioinformatics.

[26] European Centre for Disease Prevention and Control (2024). STEC infection. Annual Epidemiological Report for 2022.

[27] Pollock K.G.J., Stewart A., Beattie T.J., Todd W.T.A., Ahn C.K., Tarr P.I., Cowden J.M. (2009). From diarrhoea to haemolytic uraemic syndrome—When to seek advice. J. Med. Microbiol..

[28] Uelze L., Grützke J., Borowiak M., Hammerl J.A., Juraschek K., Deneke C., Tausch S.H., Malorny B. (2020). Typing methods based on whole genome sequencing data. One Health Outlook.

[29] Bessonov K., Laing C., Robertson J., Yong I., Ziebell K., Gannon V.P.J., Nichani A., Arya G., Nash J.H.E., Christianson S. (2021). ECTyper: In silico *Escherichia coli* serotype and species prediction from raw and assembled whole-genome sequence data. Microb. Genom..

[30] EFSA and ECDC (European Food Safety Authority and European Centre for Disease Prevention and Control) (2019). The European Union One Health 2018 Zoonoses Report. EFSA J..

[31] EFSA and ECDC (European Food Safety Authority and European Centre for Disease Prevention and Control) (2021). The European Union One Health 2019 Zoonoses Report. EFSA J..

[32] Valilis E., Ramsey A., Sidiq S., DuPont H.L. (2018). Non-O157 Shiga toxin-producing *Escherichia coli*—A poorly appreciated enteric pathogen: Systematic review. Int. J. Infect. Dis..

[33] Zweifel C., Cernela N., Stephan R. (2013). Detection of the emerging Shiga toxin-producing *Escherichia coli* O26:H11/H-sequence type 29 (ST29) clone in human patients and healthy cattle in Switzerland. Appl. Environ. Microbiol..

[34] Bielaszewska M., Mellmann A., Bletz S., Zhang W., Köck R., Kossow A., Prager R., Fruth A., Orth-Höller D., Marejková M. (2013). Enterohemorrhagic *Escherichia coli* O26:H11/H-: A new virulent clone emerges in Europe. Clin. Infect. Dis..

[35] Ogura Y., Gotoh Y., Itoh T., Sato M.P., Seto K., Yoshino S., Isobe J., Etoh Y., Kurogi M., Kimata K. (2017). Population structure of *Escherichia coli* O26:H11 with recent and repeated *stx2* acquisition in multiple lineages. Microb. Genom..

[36] Krüger A., Lucchesi P.M., Sanso A.M., Etcheverría A.I., Bustamante A.V., Burgán J., Fernández L., Fernández D., Leotta G., Friedrich A.W. (2015). Genetic characterization of Shiga toxin-producing *Escherichia coli* O26:H11 strains isolated from animal, food, and clinical samples. Front. Cell Infect. Microbiol..

[37] Leomil L., Pestana de Castro A.F., Krause G., Schmidt H., Beutin L. (2005). Characterization of two major groups of diarrheagenic *Escherichia coli* O26 strains which are globally spread in human patients and domestic animals of different species. FEMS Microbiol. Lett..

[38] Chirila F., Tabaran A., Fit N., Nadas G., Mihaiu M., Tabaran F., Cătoi C., Reget O.L., Dan S.D. (2017). Concerning Increase in Antimicrobial Resistance in Shiga Toxin-Producing *Escherichia coli* Isolated from Young Animals during 1980–2016. Microbes Environ..

[39] Tabaran A., Mihaiu M., Tăbăran F., Colobatiu L., Reget O., Borzan M.M., Dan S.D. (2017). First study on characterization of virulence and antibiotic resistance genes in verotoxigenic and enterotoxigenic *E. coli* isolated from raw milk and unpasteurized traditional cheeses in Romania. Folia Microbiol..

[40] Tabaran A., Soulageon V., Chirila F., Reget O.L., Mihaiu M., Borzan M., Dan S.D. (2022). Pathogenic *E. coli* from Cattle as a Reservoir of Resistance Genes to Various Groups of Antibiotics. Antibiotics.

[41] Aarestrup F.M. (2015). The livestock reservoir for antimicrobial resistance: A personal view on changing patterns of risks, effects of interventions and the way forward. Philos. Trans. R. Soc. Lond. B Biol. Sci..

[42] Sheikh J., Dudley E.G., Sui B., Tamboura B., Suleman A., Nataro J.P. (2006). EilA, a HilA-like regulator in enteroaggregative *Escherichia coli*. Mol. Microbiol..

[43] Miajlovic H., Smith S.G. (2014). Bacterial self-defence: How *Escherichia coli* evades serum killing. FEMS Microbiol. Lett..

[44] Burgos Y., Beutin L. (2010). Common origin of plasmid encoded alpha-hemolysin genes in *Escherichia coli*. BMC Microbiol..

[45] Toma C., Martínez Espinosa E., Song T., Miliwebsky E., Chinen I., Iyoda S., Iwanaga M., Rivas M. (2004). Distribution of putative adhesins in different seropathotypes of Shiga toxin-producing *Escherichia coli*. J. Clin. Microbiol..

[46] Okuno M., Arimizu Y., Miyahara S., Wakabayashi Y., Gotoh Y., Yoshino S., Harada T., Seto K., Yamamoto T., Nakamura K. (2023). *Escherichia* cryptic clade I is an emerging source of human intestinal pathogens. BMC Biol..

[47] Friedrich A.W., Bielaszewska M., Zhang W.L., Pulz M., Kuczius T., Ammon A., Karch H. (2002). *Escherichia coli* harboring Shiga toxin 2 gene variants: Frequency and association with clinical symptoms. J. Infect. Dis..

[48] Ethelberg S., Olsen K.E., Scheutz F., Jensen C., Schiellerup P., Enberg J., Petersen A.M., Olesen B., Gerner-Smidt P., Mølbak K. (2004). Virulence factors for hemolytic uremic syndrome, Denmark. Emerg. Infect. Dis..

[49] De Rauw K., Buyl R., Jacquinet S., Piérard D. (2018). Risk determinants for the development of typical haemolytic uremic syndrome in Belgium and proposition of a new virulence typing algorithm for Shiga toxin-producing *Escherichia coli*. Epidemiol. Infect..

[50] Wang X., Yu D., Chui L., Zhou T., Feng Y., Cao Y., Zhi S. (2024). A Comprehensive Review on Shiga Toxin Subtypes and Their Niche-Related Distribution Characteristics in Shiga-Toxin-Producing *E. coli* and Other Bacterial Hosts. Microorganisms.

[51] Escobar-Páramo P., Clermont O., Blanc-Potard A.B., Bui H., Le Bouguénec C., Denamur E. (2004). A specific genetic background is required for acquisition and expression of virulence factors in *Escherichia coli*. Mol. Biol. Evol..

[52] Gonzalez-Escalona N., McFarland M.A., Rump L.V., Payne J., Andrzejewski D., Brown E.W., Evans P.S., Croley T.R. (2013). Draft Genome Sequences of Two O104:H21 *Escherichia coli* Isolates Causing Hemorrhagic Colitis during a 1994 Montana Outbreak Provide Insight into Their Pathogenicity. Genome Announc..

[53] Mellmann A., Bielaszewska M., Köck R., Friedrich A.W., Fruth A., Middendorf B., Harmsen D., Schmidt M.A., Karch H. (2008). Analysis of collection of hemolytic uremic syndrome-associated enterohemorrhagic *Escherichia coli*. Emerg. Infect. Dis..

[54] Boisen N., Østerlund M.T., Joensen K.G., Santiago A.E., Mandomando I., Cravioto A., Chattaway M.A., Gonyar L.A., Overballe-Petersen S., Stine O.C. (2020). Redefining enteroaggregative *Escherichia coli* (EAEC): Genomic characterization of epidemiological EAEC strains. PLoS Negl. Trop. Dis..

[55] Jouve M., Garcia M.-I., Courcoux P., Labigne A., Gounon P., Le Bouguénec C. (1997). Adhesion to and invasion of HeLa cells by pathogenic *Escherichia coli* carrying the afa-3gene cluster are mediated by the AfaE and AfaD proteins, respectively. Infect. Immun..

[56] Grande L., Michelacci V., Bondì R., Gigliucci F., Franz E., Badouei M.A., Schlager S., Minelli F., Tozzoli R., Caprioli A. (2016). Whole-Genome Characterization and Strain Comparison of VT2f-Producing *Escherichia coli* Causing Hemolytic Uremic Syndrome. Emerg. Infect. Dis..

[57] Den Ouden A., Greig D.R., Rodwell E.V., Tripodo F., Olonade I., Swift C., Jenkins C. (2023). *Escherichia coli* encoding Shiga toxin subtype Stx2f causing human infections in England, 2015–2022. J. Med. Microbiol..

[58] Beutin L., Krüger U., Krause G., Miko A., Martin A., Strauch E. (2008). Evaluation of major types of Shiga toxin 2E-producing *Escherichia coli* bacteria present in food, pigs, and the environment as potential pathogens for humans. Appl. Environ. Microbiol..

[59] Liu B., Furevi A., Perepelov A.V., Guo X., Cao H., Wang Q., Reeves P.R., Knirel Y.A., Wang L., Widmalm G. (2020). Structure and genetics of *Escherichia coli* O antigens. FEMS Microbiol. Rev..

[60] Yang X., Wu Y., Liu Q., Sun H., Luo M., Xiong Y., Matussek A., Hu B., Bai X. (2021). Genomic Characteristics of Stx2e-Producing *Escherichia coli* Strains Derived from Humans, Animals, and Meats. Pathogens.

[61] Nüesch-Inderbinen M., Barmettler K., Stevens M.J.A., Cernela N. (2024). Shiga toxin-producing *Escherichia coli* isolated from hunted wild boar (*Sus. scrofa*) in Switzerland. Schweiz. Arch. Tierheilkd..

[62] Cavalcanti A.M.F., Hernandes R.T., Takagi E.H., Guth B.E.C., Ori É.L., Pinheiro S.R.S., Andrade T.S., Oliveira S.L., Cergole-Novella M.C., Francisco G.R. (2020). Virulence Profiling and Molecular Typing of Shiga Toxin-Producing *E. coli* (STEC) from Human Sources in Brazil. Microorganisms.

[63] Lindstedt B.A., Finton M.D., Porcellato D., Brandal L.T. (2018). High frequency of hybrid *Escherichia coli* strains with combined Intestinal Pathogenic *Escherichia coli* (IPEC) and Extraintestinal Pathogenic *Escherichia coli* (ExPEC) virulence factors isolated from human faecal samples. BMC Infect. Dis..

[64] De Rycke J., Milon A., Oswald E. (1999). Necrotoxic *Escherichia coli* (NTEC): Two emerging categories of human and animal pathogens. Vet. Res..

[65] Servin A.L. (2014). Pathogenesis of human diffusely adhering *Escherichia coli* expressing Afa/Dr adhesins (Afa/Dr DAEC): Current insights and future challenges. Clin. Microbiol. Rev..

[66] Gérardin J., Lalioui L., Jacquemin E., Le Bouguénec C., Mainil J.G. (2000). The afa-related gene cluster in necrotoxigenic and other *Escherichia coli* from animals belongs to the afa-8 variant. Vet. Microbiol..

